# Pediatric Non-Down Syndrome Acute Megakaryoblastic Leukemia With Unusual Immunophenotype

**DOI:** 10.7759/cureus.35965

**Published:** 2023-03-09

**Authors:** Sindhura Lakshmi Koulmane Laxminarayana, Saksham Kohli, Jhalak Agrohi, Sushma Belurkar

**Affiliations:** 1 Department of Pathology, Kasturba Medical College, Manipal, Manipal Academy of Higher Education, Udupi, IND; 2 Department of Pathology, Kasturba Medical College, Manipal, Manipal academy of Higher Education, Udupi, IND

**Keywords:** non-down syndrome acute megakaryoblastic leukemia, acute myeloid leukemia (aml), down syndrome, flow cytometry, immunophenotyping, acute megakaryoblastic leukemia

## Abstract

Acute megakaryoblastic leukemia (AMKL) is a rare subtype of acute myeloid leukemia (AML) characterized by abnormal megakaryoblasts expressing platelet-specific surface antigens. 4%-16% of childhood AMLs are AMKL. Childhood AMKL is usually associated with Down syndrome (DS). It is 500 times more common in patients with DS when compared to the general population. In contrast, non-DS-AMKL is much rarer. We describe a case of de novo non-DS-AMKL in a teenage girl child who presented with a history of excessive tiredness, fever, abdominal pain for three months, and vomiting for four days. She had lost appetite, and weight. On examination she was pale; there was no clubbing, hepatosplenomegaly or lymphadenopathy. There were no dysmorphic features or neurocutaneous markers. Laboratory tests showed bicytopenia (Hb: 6.5g/dL, total WBC count: 700/µL, platelet count: 216,000/ µL, Reticulocyte %: 0.42) and 14% blasts on the peripheral blood smear. Platelet clumps and anisocytosis were also noted. Bone marrow aspirate showed a few hypocellular particles with dilute cell trails but showed 42% blasts. Mature megakaryocytes showed marked dyspoiesis. Flow cytometry on bone marrow aspirate showed myeloblasts and megakaryoblasts. Karyotyping showed 46 XX. Hence, a final diagnosis of non-DS-AMKL was established. She was treated symptomatically. However, she was discharged on request. Interestingly, the expression of erythroid markers such as CD36 and lymphoid markers like CD7 is usually seen in DS-AMKL and not in non-DS-AMKL. AMKL is treated with AML-directed chemotherapies. Although complete remission rates are similar to other AML subtypes, overall survival is only about 18-40 weeks.

## Introduction

Acute megakaryoblastic leukemia (AMKL) is a rare subtype of acute myeloid leukemia (AML) with 20% or more blasts and at least half of them being of the megakaryocytic lineage [1]. AMKL is most frequently associated with Down syndrome (DS) but can also arise de novo or may be secondary to chemotherapy and myelodysplastic syndrome progression [2]. AMKL comprises only 1% of the adult AML population, whereas in childhood AML cases, its incidence is in the range of 4% to 16% [3].

AMKL occurs more commonly in DS children as opposed to those without DS; non-DS-AMKL and adult-AMLK account for <1% of all AMKL individuals [4]. According to a comparison study conducted by Asahito Hama, the 10-year overall survival estimate was 79% for DS-AMKL, and 76% for non-DS-AMKL patients, with a median follow-up of 78 months [5].

AMKL is characterized by extensive myelofibrosis, making bone marrow aspiration difficult. The bone marrow biopsy examination for morphology and immunohistochemistry helps in arriving at an accurate diagnosis in cases of dry tap.

With recent advancements in genomic sequencing, some chimeric oncogenes have been identified in a substantial number of non-DS-AMKL cases, including* RBM15-MKL1, CBFA2T3-GLIS2, KMT2A* gene rearrangements, and *NUP98-KDM5A*. However, the etiology of 30-40% of cases still remains unclear [6]. We herein present one such case of AMKL in a child without DS.

## Case presentation

A 13-year-old girl presented with complaints of fever and abdominal pain, for three months and vomiting for four days. The fever was gradual in onset, intermittent in nature, low-grade, relieved on medication, and associated with chills with increased intensity for four days. Vomiting was non-bilious, contained food particles, non-projectile in nature. Abdominal pain was diffuse, with no history of radiation. The pain was relieved by vomiting. She complained of loss of appetite, weight loss, and fatigue. On general examination, the patient was alert and active, with a blood pressure of 110/70mm of Hg, pulse rate of 90 beats per minute, respiratory rate of 18 per min, and SpO_2_ of 96% at room air. The body mass index was found to be 14.3 kg/m^2^. A head-to-toe examination revealed pallor, muscle wasting, cachexia, and dry lusterless skin. There was no icterus, cyanosis, digital clubbing, lymphadenopathy or edema. A review of the systemic examination was unremarkable.

Investigations

The child had severe anemia and leucopenia, with 14% blasts in the peripheral smear. Serum lactate dehydrogenase (LDH) and erythrocyte sedimentation rate (ESR) were found to be elevated, with a low reticulocyte count and a normal uric acid level (Table 1). In view of the fever, a blood culture was sent which was unremarkable for any active infection. In light of her cytopenia with a low reticulocyte count and circulating blasts, bone marrow examination for morphology and immunophenotyping was performed.

**Table 1:** Summary of laboratory investigations ESR - Erythrocyte sedimentation rate, LDH - Lactate dehydrogenase

	Observed Value	Reference Range
Hemoglobin	6.5 g/dL	12-15 g/dL
Hematocrit	19.4%	34%-46%
Reticulocyte Count	0.42%	0.5%-2.5%
ESR	>140 mm/hr	0-20 mm/hr
Total Leucocyte Count	0.7 x 10^3^/µL	4-10 X 10^3^/µL
Lymphocytes	85%	18%-44%
Neutrophils	1%	42%-74%
Abnormal cells/blasts in peripheral blood	14%	Absent
Platelet Count	216 x 10^3^/µL	150-400 x 10^3^/µL
LDH	313 U/L	125-220 U/L

**Figure 1:**
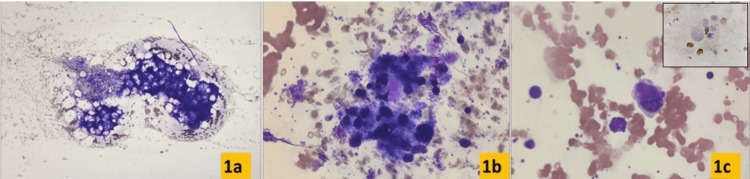
Bone marrow aspirate, Wright stain. (a-c) 4x, 10x, and 40x, respectively. (a) Hypocellularity and dilute cell trails. (b, c) Dyspoietic micromegakaryocytes and blasts. Inset in (c) shows myeloperoxidase negative blasts.

The initial bone marrow examination from right ileac crest failed and yielded hemodilute marrow. Hence, the procedure was repeated from left side. The morphologic examination of the aspirate smears revealed a few marrow particles with reduced cellularity for age with dilute cell trails. A nucleated cell differential count revealed 42% blasts. Auer rods were not seen. Megakaryocytes showed marked dyspoiesis with hypolobation and hypogranularity, micromegakaryocytes were present. Myeloperoxidase (MPO), periodic acid-Schiff (PAS), and Sudan Black B (SBB) were negative in blasts (Figures 1a-1c).

**Figure 2:**
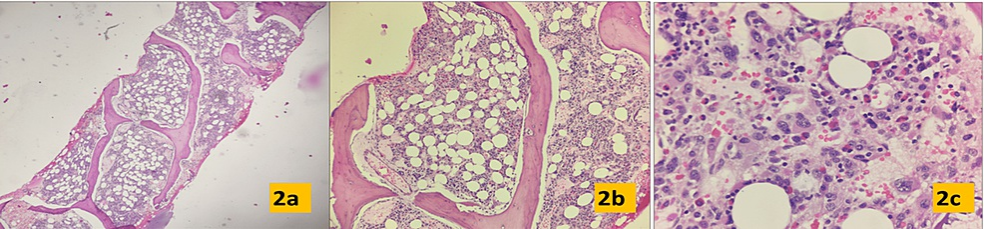
Bone marrow biopsy, Hematoxylin and eosin. (a-c) 4x, 10x, and 40x, respectively. The image illustrates the reduced marrow cellularity and increased number of bizarre blasts.

Bone marrow biopsy showed hypocellular marrow with decreased cellularity for age. Normal hematopoiesis was markedly suppressed due to infiltration by abnormal blasts. They were large cells with intermediate nucleo-cytoplasmic ratio with irregular and vesicular nuclei, irregular nuclear contours, anisonucleosis with the peri-sinusoidal distribution. Intra-sinusoidal megakaryopoiesis was present, and scattered lymphoid cells and eosinophils were seen. There was grade 1 reticulin fibrosis (Figures 2a-2c).

**Figure 3:**
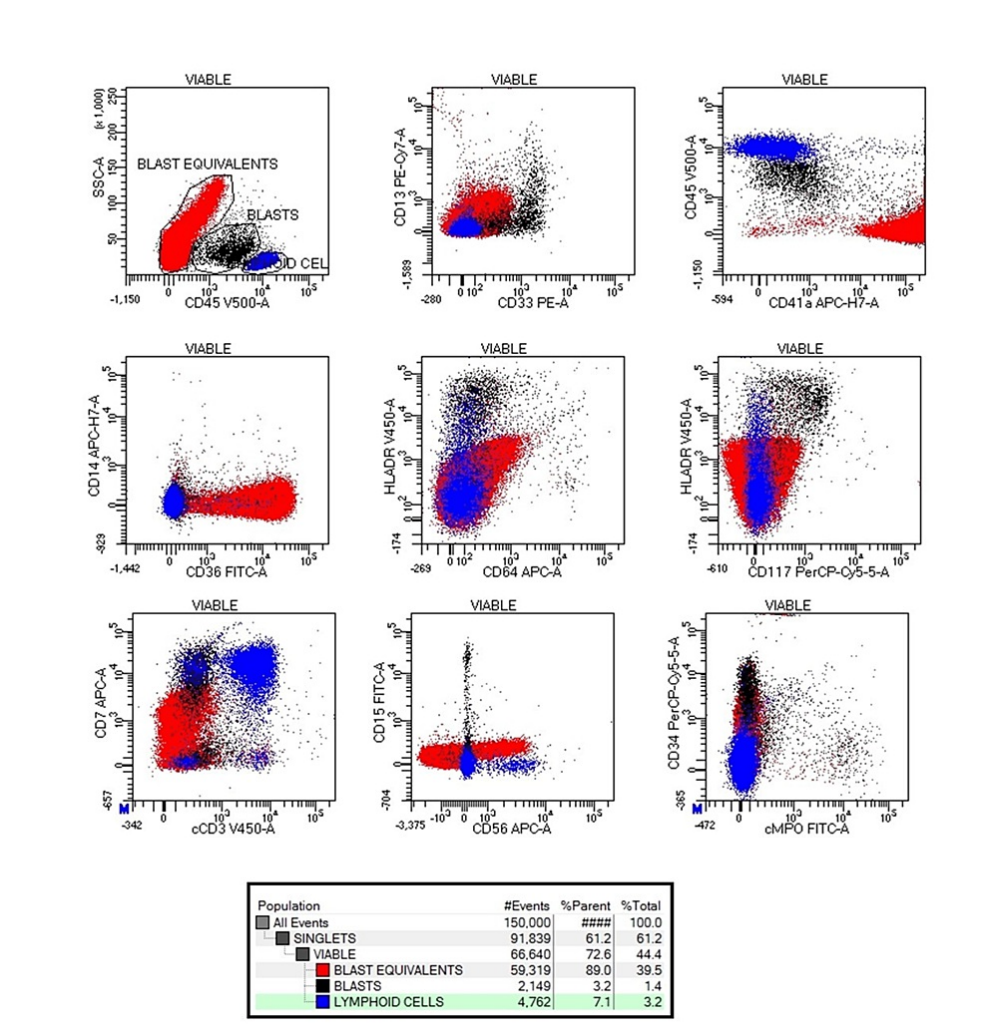
Flow cytometry on bone marrow aspirate. Both myeloblasts highlighted in black (positive for CD13, CD33 and CD117) and megakaryoblasts highlighted in red (positive for CD41, CD36) were positive for CD7, CD56, CD34 and HLADR. Normal T lymphoid cells are depicted in blue.

Immunophenotyping was performed on bone marrow aspirate with low side scatter characteristics (SSC) and moderate CD45+ events gated as blasts (3.2% of viable events) and low to intermediate SSC CD45 negative events gated as blast equivalents (89.0% of viable events). Blasts were positive for CD33 (heterogeneous, moderate), CD13 (heterogeneous, dim), CD117 (homogeneous, dim), CD34 (homogeneous, moderate), HLADR (homogeneous, bright), and CD7 (homogeneous, moderate). Blast equivalents were positive for CD41 (homogeneous, bright), CD36 (homogeneous, bright), CD34 (homogeneous, moderate), HLADR (heterogeneous, dim), CD56 (heterogeneous, moderate) and CD7 (homogeneous moderate). Normal lymphoid cells were considered as internal controls for defining the expression pattern. Hence, a final diagnosis of non-DS-AMKL was established (Figure 3).

**Figure 4:**
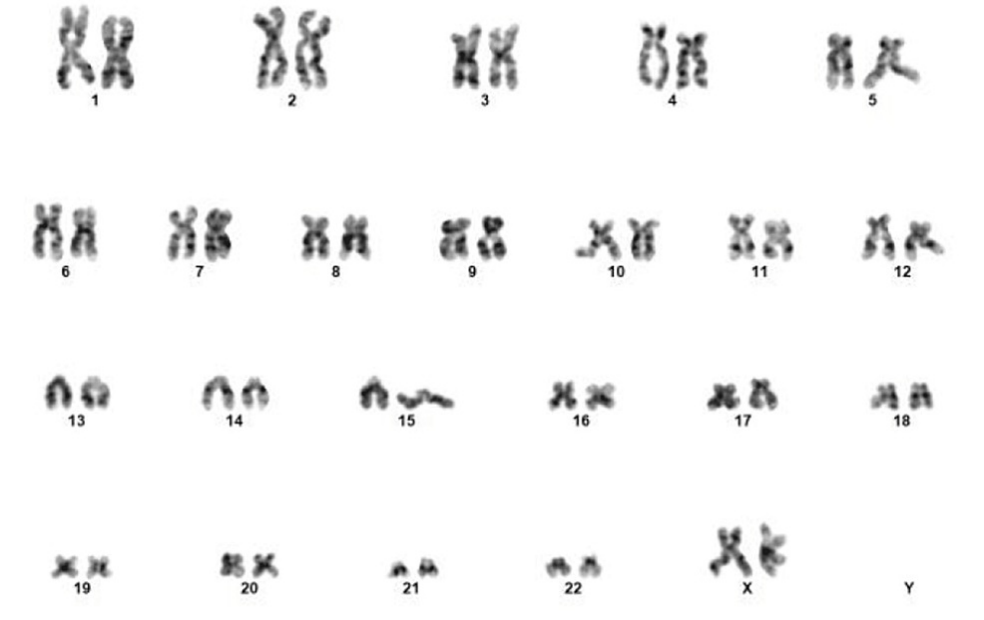
Conventional karyotyping on bone marrow showed normal diploid female karyotype 46 XX

Conventional karyotype analysis revealed normal diploid female karyotype 46, XX in all 20 cells (Figure 4).

Fluorescent in-situ hybridization and molecular testing could not be performed as the child’s parents sought discharge.

Treatment/course in the hospital

While waiting for the biopsy reports, the child had persistent fever spikes for which she was started on oral antibiotics Amoxicillin and Co-trimoxazole for four days, and oral hydration. She was also transfused with one unit-packed RBC 250 mL in view of anemia. The definitive plan was to start the patient on chemotherapy; however, the parents were unwilling to continue treatment and were hence discharged at request.

## Discussion

Non-DS-AMKL has been known to have a grave prognosis when compared to AMKL in patients with DS. This could possibly be attributed to the presence of mutations in the transcription factor GATA1 in the latter, which renders the tumor sensitive to reduced-intensity chemotherapeutic agents [7-13]. This could also provide a plausible explanation for the better overall survival (OS) of non-Down Syndrome AMKL patients with an acquired trisomy 21 and GATA1 mutation. According to AML-BFM 04 trial, CD7 surface marker expression was found to be very predictive of GATA1 mutation [14].

The current diagnostic modalities for AMKL are bone marrow evaluation for morphology, immunophenotyping by flow cytometry on bone marrow aspirate or peripheral blood, and immunohistochemistry on bone marrow biopsy. Although it is difficult to diagnose AMKL solely by morphological features, the presence of cytoplasmic blebbing, platelet budding, and clustering of blasts supports the diagnosis. Bone marrow is usually fibrotic and hence difficult to aspirate, as was in our case [1]. The megakaryoblasts are reported to be large-sized cells with an increased nucleo-cytoplasmic ratio. Often, excessively vacuolated and scanty basophilic cytoplasm is seen.

Megakaryoblasts typically stain negative for MPO, Sudan black, and alpha naphthyl butyrate esterase with variable activity for alpha naphthyl acetate esterase and PAS diastase stain [15]. 

According to the WHO classification, there are 4 categories of AML: AML with recurrent genetic abnormalities, AML with myelodysplasia-related changes, AML not otherwise specified (NOS), and myeloid proliferations associated with DS. There are 8 subgroups under the AML NOS category based on maturation. AML NOS with megakaryocytic differentiation or AMKL is characterized by varied biology and may present with a wide range of clinical manifestations. In addition, other subgroups of AML may also show megakaryocytic differentiation. For example, 70%-90% of patients with DS and AML show megakaryocytic differentiation. Ten percent of neonates with DS develop transient abnormal myelopoiesis which shows megakaryocytic differentiation. Recurrent t(1;22) (RBM15-MKL1) is also associated with neonatal AMKL. A new subtype with “RAM immunophenotype,” which correlates with CBFA2T3::GLIS2, also shows megakaryocytic differentiation [16]. Immunophenotyping is essential to detect megakaryocytic differentiation (at least one or more of the platelet glycoproteins: CD41 (glycoprotein llb), CD61 (glycoprotein IIIa), or CD42b (glycoprotein lb). In addition, stem/progenitor cell markers (CD34, CD33, and CD117), erythroid/megakaryocytic/monocytic markers (CD36), and T-cell markers (e.g., CD7) are also required for accurate immunophenotyping of AMKL.

The EuroFlow study of the immunophenotypic profile of 72 AMKL and 114 non-AMKL AML demonstrated the expression of CD42b (odds ratio [OR]: 119), CD42a.CD61(OR: 103), CD41(OR: 15), CD71(OR: 7.0), and CD7(OR: 5.2) could accurately discriminate AMKL from non-AMKL AML. Lack of expression of HLADR (seen only in 39% of AMKL), CD15, and CD13 (seen only in 23% of AMKL) was also a prominent feature [15]. This study also showed a higher frequency of CD34+/CD117+ blasts in TAM and AML associated with DS patients as compared to AMKL-NOS. Similar findings were also reported by Hama et al. They detected CD7 in 88% of DH-AMKL and 53% of non-DS-AMKL [5]. In the present case, blast equivalents showed homogeneous bright positivity for CD41, and CD36 and moderate positivity for CD7. In addition, they also expressed CD34 while being negative for CD117. On the contrary, a dim expression of HLADR and CD13 was noted. It has been noted that normal platelets may piggyback on the blasts which may be falsely interpreted as megakaryocytic differentiation, particularly in the case of MPO-negative AML such as monocytic leukemia. In such instances assessment of cytoplasmic megakaryocytic markers may aid in the diagnosis. However, this practice has not been recommended by large studies [15,16]. Indeed, the EuroFlow study demonstrated that a panel of backbone markers CD34, CD117, HLADR, and CD45 with CD13, and megakaryocytic markers confirmed AMKL diagnosis when there was a high degree of suspicion based on morphological and/or clinical criteria [15].

In contrast to DS-AMKL, leukemic cells carry only megakaryocytic cell-surface markers without any erythroid markers. Blasts in DS-AMKL are more likely to express CD7 and CD11b than those in non-DS-AMKL and adult-AMKL. They are more likely to express CD13, CD33, and CD36 than non-DS pediatric AMKL, and show greater CD56 expression compared to adult AMKL [5,14-16]. However, in the present case, the blasts expressed myeloid, lymphoid, and platelet/erythroid/monocytic markers despite the absence of phenotypic features of Down syndrome in the child.

A significant proportion of non-DS-AMKL carries chimeric oncogenes including *RBM15-MKL1, CBFA2T3-GLIS2, NUP98-KDM5A,* and *MLL* gene rearrangements. The most common genetic abnormalities are *KMT2A::MLLT3* and *KMT2A::MLLT10*, which show megakaryoblastic differentiation and are associated with low blast count in the bone marrow. Megakaryoblastic morphology might also be seen with inversion (3) and t(3;3), but all of these fall under AMKL with recurrent genetic abnormalities. However, molecular characterization could not be performed in the present case.

The treatment is divided into induction therapy to achieve morphological remission and minimal residual disease at flow cytometric analysis, followed by post-remission or consolidation therapy. Treatment of AMKL is heavily dependent on risk-stratification of patients which has been constantly changing over time, especially after the development of advancement in diagnostic modalities to identify recurrent and cryptic cytogenetic aberrations, which was not possible with the conventional karyotyping.

Most of the studies agree on the fact that the high-risk group comprises monosomy 7, *NUP98-KDM5 A, CBFA2T3-GLIS2*, 9p anomalies including *KMT2A/MLLT3,* -13/13q and -15; whereas the low-risk group includes abnormalities in 7p [17,18]. Interestingly, a study conducted by Schweitzer did not find t(1,22)(p13;q13) and monosomy 7 to have a bad prognosis. However, it should be noted that treatment response characterized by bone marrow morphology on day 15 and day 28 is an independent prognostic marker [18]. The outcome is generally poor, with lower event-free survival than DS-AMKL and pediatric AML, even in the face of intensified treatment. CBFA2T3-GLIS2 is the most frequent chimeric oncogene identified to date in this subset of patients and confers a poor prognosis.

Although there are a lot of schools of thought regarding the role of allogeneic hematopoietic stem cell transplant (HSCT), the current consensus acknowledges the best strategy to opt for intensive chemotherapy, followed by HSCT in the first complete remission (CR1) for the high-risk group, whereas HSCT should be reserved for patients who are refractory to induction chemotherapy in the low-risk group. Although complete remission rates are similar to other AML subtypes, overall survival is only about 18-40 weeks [18].

## Conclusions

Non-DS-AMKL is a rare subtype of AMKL with varied clinical and immunophenotypic profiles. It is important to identify AMKL even in non-DS children due to its worse prognosis. Immunophenotyping is essential for the accurate detection of megakaryocytic differentiation.
